# Risk factors for hypoxaemia following hip fracture surgery in elderly patients who recovered from COVID-19: a multicentre retrospective study

**DOI:** 10.3389/fmed.2023.1219222

**Published:** 2023-07-11

**Authors:** Wen Chi, Peng Pang, Zhenguo Luo, Xiaobing Liu, Wenbo Cai, Wangyang Li, Jianhong Hao

**Affiliations:** ^1^Department of Operating Room, HongHui Hospital, Xi’an JiaoTong University, Xi’an, China; ^2^Department of Anaesthesiology, Binzhou Medical College Affiliated Hospital, Binzhou, China; ^3^Department of Anaesthesiology, HongHui Hospital, Xi’an JiaoTong University, Xi’an, China; ^4^Department of Orthopedic, Linfen Hospital Affiliated to Shanxi Medical University, Linfen, China

**Keywords:** COVID-19, hypoxia, hip fractures, aged, risk

## Abstract

**Objectives:**

To explore the risk factors associated with postoperative hypoxaemia in elderly patients who have recovered from coronavirus disease (COVID-19) and underwent hip fracture surgery in the short term.

**Design:**

Multicentre retrospective study.

**Setting:**

The study was performed in three first 3A-grade hospitals in China.

**Participants:**

A sequential sampling method was applied to select study participants. Medical records of 392 patients aged ≥65 years who had recovered from COVID-19 and underwent hip fracture surgery at three hospitals in China between 1 November, 2022, and 15 February, 2023, were reviewed.

**Interventions:**

Patients were assigned to hypoxaemia or non-hypoxaemia groups, according to whether hypoxaemia occurred after surgery. Univariate and multivariate logistic regression analyses were used to identify independent risk factors for postoperative hypoxaemia.

**Results:**

The incidence of postoperative hypoxaemia was 38.01%. Statistically significant differences were found between the two groups in terms of age, body mass index (BMI), American Society of Anesthesiologists (ASA) classification, presence of expectoration symptoms, preoperative hypoxaemia, chronic obstructive pulmonary disease, pulmonary inflammation, time between recovery from COVID-19 and surgery, anaesthetic mode, surgical procedure, intraoperative blood loss, intraoperative infusion, duration of surgery, and length of hospital stay (*p* < 0.05). Furthermore, patients with BMI ≥28.0 kg/m^2^, expectoration symptoms, presence of preoperative hypoxaemia, ASA classification III, time between recovery from COVID-19 and surgery ≤2 weeks, and general anaesthesia were potential risk factors for postoperative hypoxaemia.

**Conclusion:**

Obesity, expectoration symptoms, preoperative hypoxaemia, ASA classification III, time between recovery from COVID-19 and surgery ≤2 weeks, and general anaesthesia were potential risk factors for postoperative hypoxaemia in elderly patients who recovered from COVID-19 and underwent hip fracture surgery in the short term.

## 1. Introduction

Currently, the global coronavirus disease (COVID-19) pandemic is ongoing (1). However, compared to the early days of the pandemic, most patients with COVID-19 exhibit mild symptoms and recover after 2 weeks. The effect of COVID-19 on perioperative management remains a major concern (2). Research has shown that patients with COVID-19 are at a higher risk of postoperative respiratory failure (3–5). Despite a growing body of literature on COVID-19, there is limited research on identifying perioperative risk factors in recently recovered patients who undergo surgery in a short term, especially, in elderly patients with multiple comorbidities; these patients are at a higher risk of perioperative complications.

Hip fractures are a common occurrence in elderly patients, and surgical intervention is often required. With the aging of the Chinese population, the number of patients undergoing hip fracture surgery is increasing (6, 7). Postoperative hypoxaemia is common in patients with hip fractures and is associated with prolonged hospital stays, high costs, and increased mortality (8). At present, research on postoperative hypoxaemia in elderly patients with hip fracture mainly focuses on patients with or without COVID-19, and research on postoperative hypoxaemia in elderly patients with hip fracture who have recovered from COVID-19 is lacking (9, 10). We hypothesized that factors such as basic characteristics of patients, COVID-19-related sequelae, and changes in blood indicators would affect the development of postoperative hypoxaemia in elderly patients who have recovered from COVID-19 and underwent hip fracture surgery. To test this hypothesis, we conducted a multicentre retrospective study to collect and analyse the relevant risk factors associated with postoperative hypoxaemia in elderly patients who have recovered from COVID-19 and underwent hip fracture surgery in the short term.

## 2. Materials and methods

### 2.1. Design, sample, and criteria for participation

This study was approved by our institutional review board. In this retrospective study, only medical records and case information were analysed, no patients were directly involved in setting the research questions or the outcome measures, and patient information was anonymized; therefore, written inform consent was waived by ethic committee.

This multicentre retrospective study included 392 patients who underwent hip fracture surgery at three hospitals (HongHui Hospital, Xi’an JiaoTong University, Xi’an, Shaanxi Province; Binzhou Medical College Affiliated Hospital, Binzhou, Shandong Province; and Linfen Hospital Affiliated to Shanxi Medical University, Linfen, Shanxi Province) in China between 1 November, 2022, and 10 February, 2023.

The sample size was determined based on the following assumptions. According to a previous study in Shanghai, China (9), we used a sample proportion of 30.23, 95% confidence interval, and margin of error of 0.05. The sample size was calculated using the following formula:


$$\begin{array}{c} N=z^{2}p\left(1-p\right)/d^{2}n\\ ={\left(1.96\right)}^{2}\times \left(0.3023\right)\;\left(1-0.3023\right)/{\left(0.05\right)}^{2}=325. \end{array}$$

where, *n* = sample size; *p* = 30.23% of proportion; *q* = 1 − *p*; *d* = desired degree of precision; and *Z* = the standard normal value at 95% confidence level. Considering a non-response rate of 5%, we increased the sample size to 342: *n* = (325 + 17) = 342. To obtain more convincing results, we further expanded the sample size by 50, for a total of 392 samples.

**Figure 1:**
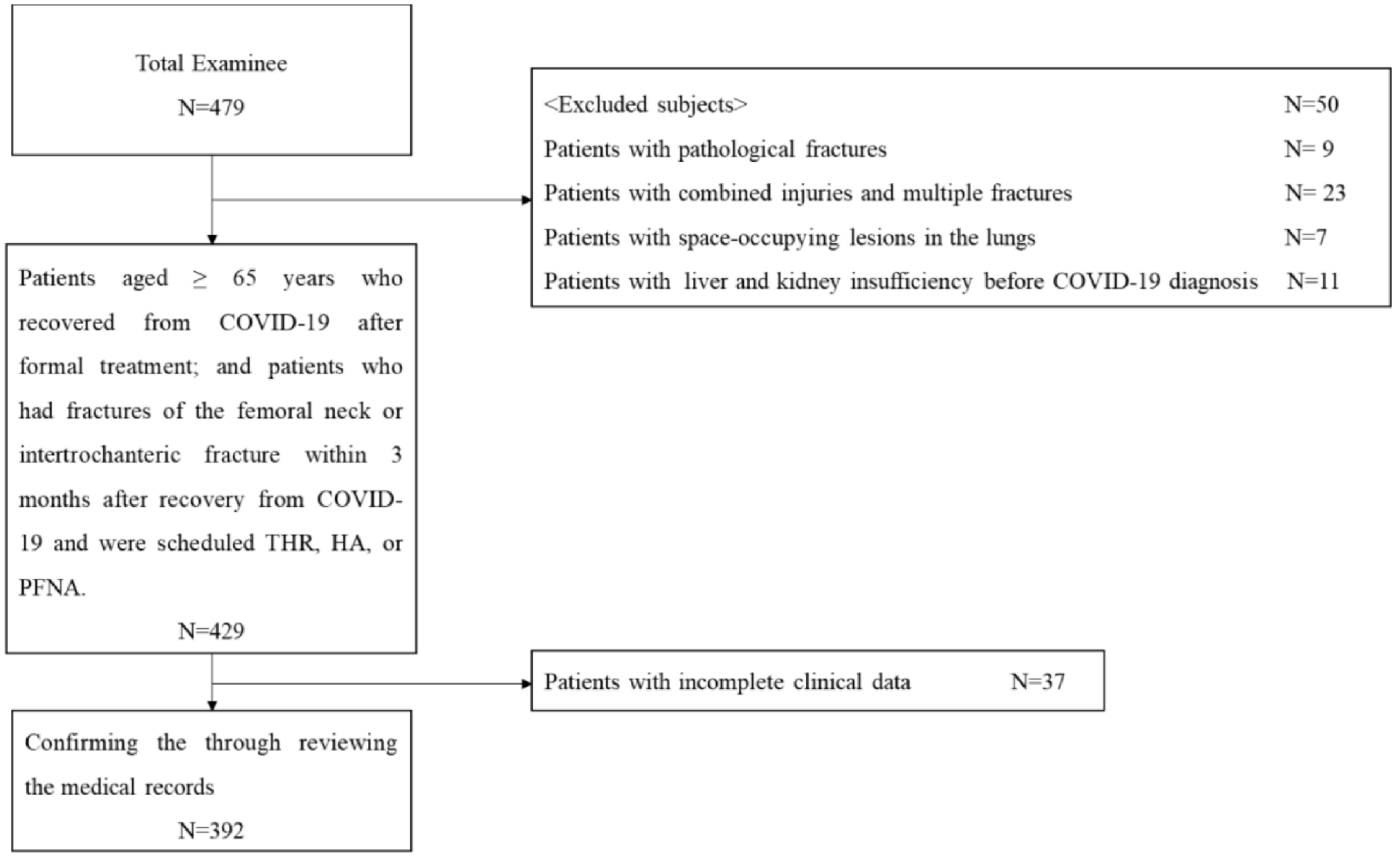
Process of selecting the study subjects.

Inclusion criteria were as follows: (1) patients aged ≥65 years who recovered from COVID-19 after formal treatment; and (2) patients who had fractures of the femoral neck or intertrochanteric fracture within 3 months after recovery from COVID-19 and were scheduled for total hip replacement (THR), hemiarthroplasty (HA), or proximal femoral nail antirotation (PFNA). The exclusion criteria were as follows: (1) combined injuries and multiple fractures; (2) pathological fractures; (3) space-occupying lesions in the lungs; (4) liver and kidney insufficiency before COVID-19 diagnosis; and (5) patients with incomplete clinical data. The selection process is illustrated in Figure 1.

### 2.2. Data collection

**Figure 2:**
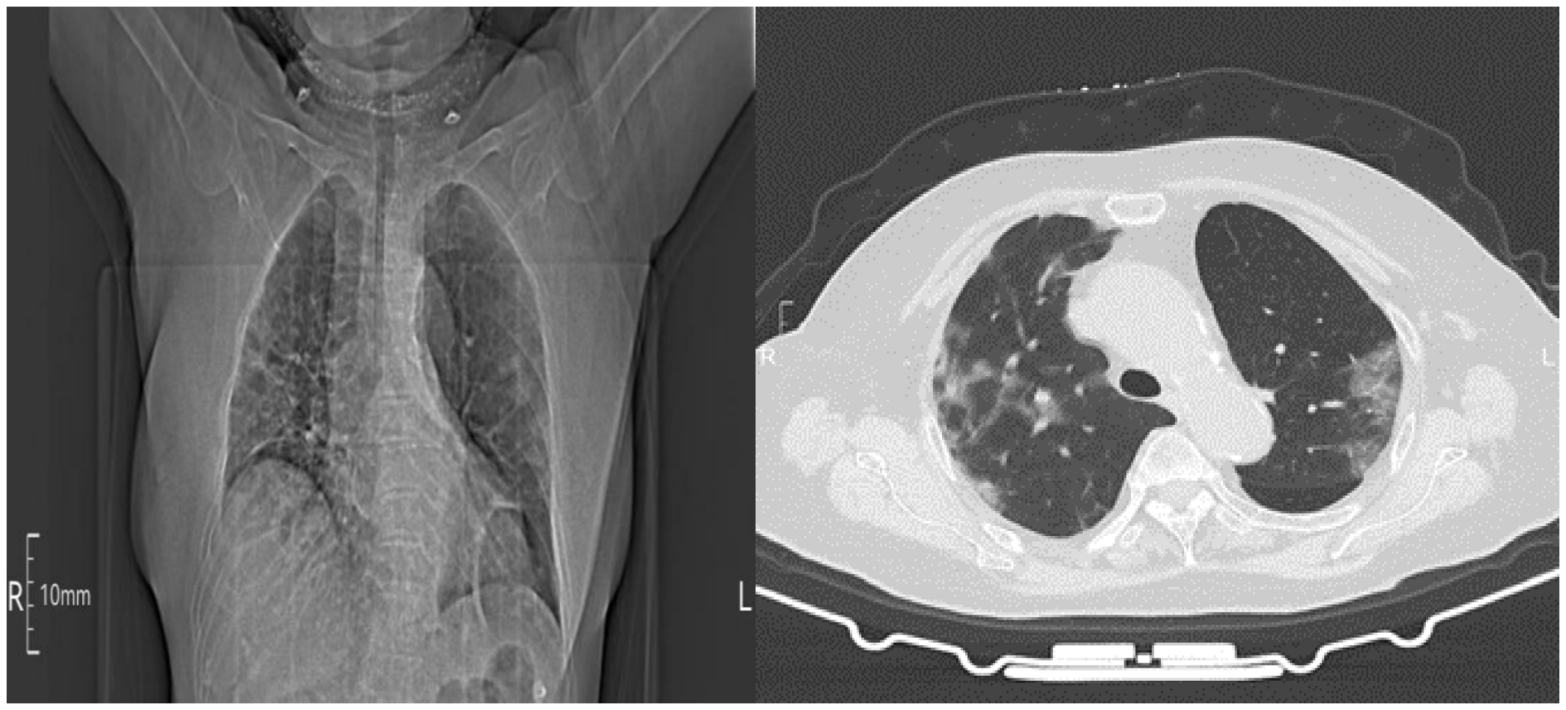
Chest computed tomography (CT) indicated non-regional ground-glass opacities in the lungs and infiltrative shadows.

Data were collected using a structured checklist adapted from different studies (9, 11, 12). The tool was prepared in English, and data were extracted through a review of patients’ medical charts by trained data collectors. The data extracted included age, sex, height, weight, symptoms including cough, expectoration, chest tightness, and hypoxaemia; American Society of Anesthesiologists (ASA) classification; comorbidities including hypertension, diabetes, coronary heart disease, chronic obstructive pulmonary disease (COPD), stroke, smoking status, chest computed tomography (CT) scans, time between recovery from COVID-19 and surgery, time from admission to surgery, fracture site (fracture of femoral neck or intertrochanteric fracture), surgical procedure (THR, HA, and PFNA), anaesthetic mode (general anaesthesia or intraspinal anaesthesia), intraoperative variables including blood loss, infusion, and duration of surgical time; length of hospital stay; and laboratory parameters including leukocytes, neutrophils, lymphocytes, haemoglobin, glutamic-pyruvic transaminase, glutamic-oxaloacetic transaminase, urea, creatinine, and lactic dehydrogenase. To ensure data quality, chart review was done on 5% (*n* = 20) of the study population to test the checklist’s structure and completeness, and essential modifications were made at HongHui Hospital, Xi’an JiaoTong University. Preoperative hypoxaemia was defined as an arterial partial pressure of oxygen to fraction of inspired oxygen (PaO_2_/FiO_2_) ratio of ≤300. Postoperative hypoxaemia was defined as a pulse oxygen saturation <94% on room air (13–15). Patients were sequentially assigned to hypoxaemia or non-hypoxaemia groups, according to whether hypoxaemia occurred after surgery. Chest-CT scans were used to determine the presence of pulmonary inflammation (Figure 2).

### 2.3. Statistical analysis

All data were analysed using SPSS^®^ for Windows (version 18; SPSS Inc., Chicago, IL, United States). The measurement data of clinical indices are expressed as the mean ± standard deviation (SD) and analysed using independent sample *t*-tests or the Wilcoxon rank-sum test. Count data are expressed as numbers (percentages) and analysed using either the chi-square test or Fisher’s exact test, depending on the sample size and distribution of data. Factors with a *p* < 0.05 in the univariate analysis were selected for logistic multivariate regression analysis. Statistical significance was set at *p* < 0.05.

## 3. Results

### 3.1. Patients’ demographic, clinical, and laboratory characteristics

A total of 392 patients were included, including 301 (76.78%) women and 91 (23.22%) men, with an average age of 79.32 years (SD, 7.97; range, 65–95) and an average body mass index (BMI) of 23.43 kg/m^2^ (SD, 3.59; range, 14.17–39.52). Of the 392 patients, 208 had femoral neck fractures, and 184 had intertrochanteric fractures.

**Figure 3:**
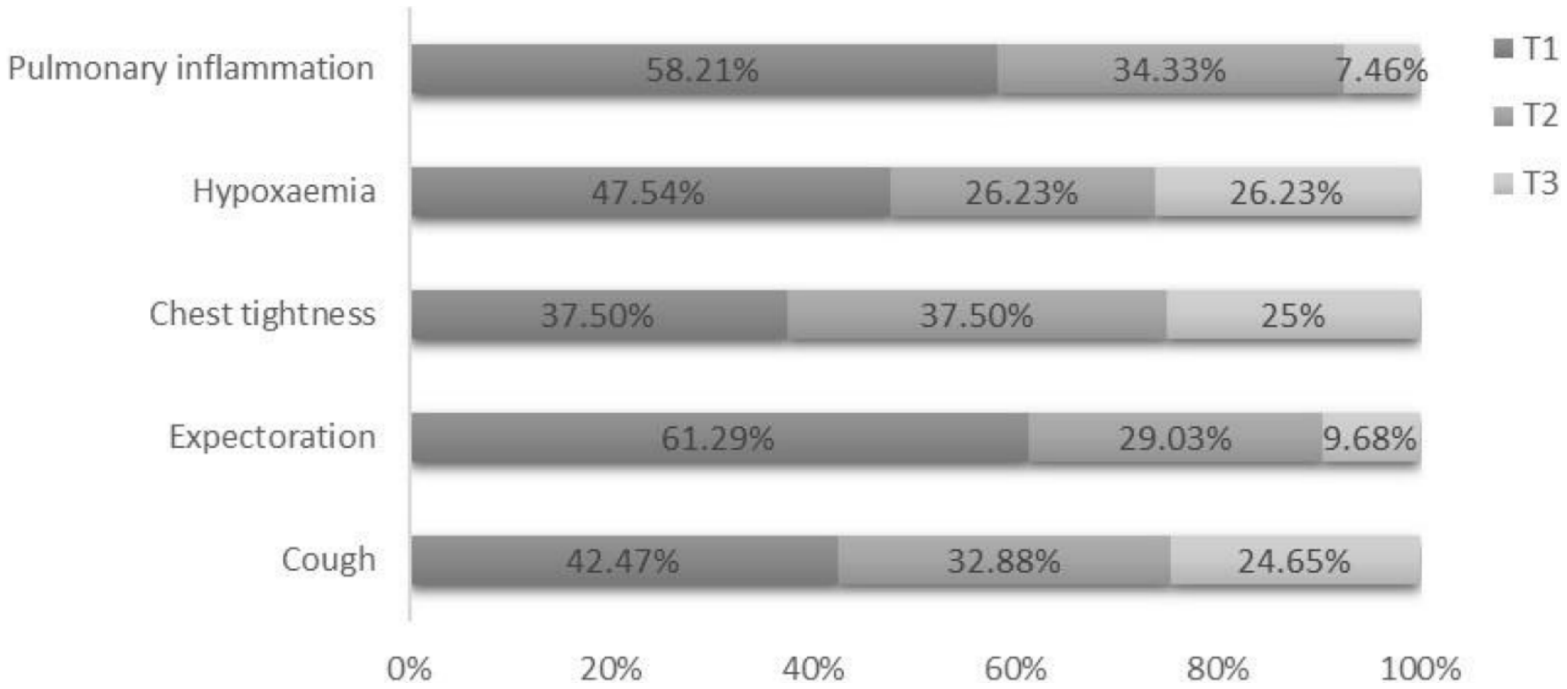
Proportion of patients experiencing cough, expectoration, chest tightness, hypoxaemia, and pulmonary inflammation at different time intervals. T1: time between recovery from COVID-19 and surgery ≤2 weeks; T2: 2 weeks < time between recovery from COVID-19 and surgery ≤4 weeks; T3: time between recovery from COVID-19 and surgery >4 weeks.

Regarding symptoms, 18.62% experienced cough, 7.90% expectoration, 6.12% chest tightness, and 15.56% hypoxaemia. Among 392 patients, 17.09% experienced pulmonary inflammation (Table 1). A high proportion of patients experienced cough, expectoration, hypoxaemia, and pulmonary inflammation within 2 weeks of recovery from COVID-19 (Figure 3).

**Table 1:** Demographic and clinical characteristics of the patients.

Variables	Hypoxaemia	Non-hypoxaemia	Overall	*p*
Number	149 (38.01)	243	392	
Age (years)				<0.001^*^
65–74	31 (20.81)	88 (36.21)	119 (30.36)	
75–84	52 (34.90)	109 (44.86)	161 (41.07)	
≥85	66 (44.29)	46 (18.93)	112 (28.57)	
Sex				0.055
Women	99 (66.44)	202 (83.13)	301 (76.78)	
Men	50 (33.56)	41 (16.87)	91 (23.22)	
BMI (kg/m^2^)				0.022^*^
18.5–23.9	59 (39.60)	136 (55.97)	195 (49.74)	
<18.5	17 (11.41)	21 (8.64)	38 (9.69)	
24.0–27.9	49 (32.89)	72 (29.63)	121 (30.88)	
≥28.0	24 (16.10)	14 (5.76)	38 (9.69)	
Symptoms				
Cough	21 (14.09)	52 (21.39)	73 (18.62)	0.431
Expectoration	19 (12.75)	12 (4.93)	31 (7.90)	0.011^*^
Chest tightness	11 (7.38)	13 (5.34)	24 (6.12)	0.227
Hypoxaemia	48 (32.21)	13 (5.34)	61 (15.56)	<0.001^*^
ASA classification				<0.001^*^
I	18 (12.08)	52 (21.40)	70 (17.86)	
II	43 (28.86)	76 (31.28)	119 (30.36)	
III	88 (59.06)	115 (47.32)	203 (51.78)	
Comorbidities				
Hypertension	31 (20.80)	48 (19.75)	79 (20.15)	0.802
Diabetes	30 (20.13)	54 (22.22)	84 (21.42)	0.501
Coronary heart disease	33 (22.14)	60 (24.69)	93 (23.72)	0.683
Stroke	22 (14.76)	31 (12.75)	53 (13.52)	0.241
COPD	28 (18.79)	30 (12.34)	58 (14.79)	0.048^*^
Smoking status	16 (10.73.)	28 (11.52)	44 (11.22)	0.148
Pulmonary inflammation	40 (26.84)	27 (11.11)	67 (17.09)	0.011^*^
Time between recovery from COVID-19 and surgery (weeks)				<0.001^*^
≤2	47 (31.54)	30 (12.35)	77 (19.64)	
2–4	31 (20.81)	57 (23.46)	88 (22.45)	
>4	71 (47.65)	156 (64.19)	227 (57.91)	
Time from admission to surgery (hours)	39.81 ± 3.78	41.49 ± 4.58	40.73 ± 5.08	0.725
Fracture site				0.071
Fracture of femoral neck	74 (49.66)	134 (55.14)	208 (53.06)	
Intertrochanteric fracture	75 (50.34)	109 (44.86)	184 (46.94)	
Surgical procedure				<0.001^*^
THR	23 (15.44)	59 (24.28)	82 (20.92)	
HA	85 (57.05)	124 (51.03)	209 (53.32)	
PFNA	41 (27.51)	60 (24.69)	101 (25.76)	
Anesthetic mode				
General anesthesia	96 (64.43)	105 (43.21)	201 (51.28)	<0.001^*^
Intraspinal anesthesia	53 (35.57)	138 (56.79)	191 (48.72)	
Variables of intra-operation				
Blood loss (mL)	271.80 ± 42.55	314.80 ± 37.95	301 ± 33.91	0.031^*^
Infusion (mL)	1690.70 ± 249.60	1909.50 ± 245.80	1879 ± 225.31	0.012^*^
Duration of surgical time (min)	74.65 ± 3.84	87.52 ± 3.63	82.79 ± 2.41	0.044^*^
Length of hospital stay, days	11.32 ± 1.14	8.56 ± 2.87	10.71 ± 1.97	0.011^*^

BMI, body mass index; COPD, chronic obstructive pulmonary disease; ASA, American Society of Anesthesiologists; COVID-19, novel coronavirus disease 2019; THR, total hip replacement; HA, hemiarthroplasty; PFNA, proximal femoral nail antirotation. ^*^p < 0.05.

Regarding laboratory parameters for the 392 patients, leukocytes count, neutrophil count, glutamic-pyruvic transaminase level, glutamic-oxaloacetic transaminase level, blood urea level, serum creatinine level, and lactic dehydrogenase level were above the upper limit of normal in 5.35%, 2.80%, 3.06%, 5.61%, 5.86%, 7.65%, and 7.90% of patients, respectively. The lymphocyte count was below the lower limit of normal in 10.96% of the patients.

### 3.2. Incidence of postoperative hypoxaemia

The incidence of postoperative hypoxaemia was 38.01% (Table 1). Of all postoperative hypoxaemia cases, 92.62% (138 of 149) presented with an oxygen saturation of 85%–93% on room air; these patients returned to the ward after surgery for supplemental oxygen therapy, and 7.38% (11 of 149) presented with an oxygen saturation of <85% on room air. After surgery, all patients were administered general anaesthesia, kept intubated with an endotracheal tube, and were subsequently admitted to the intensive care unit (ICU) for further treatment. In the ICU, one patient died on postoperative day 17 due to respiratory failure. In the ward, one patient without postoperative hypoxaemia died on postoperative day 2, likely due to pulmonary embolism. All the other patients recovered and were discharged.

### 3.3. Risk factors for postoperative hypoxaemia

Based on age, patients were divided into three groups: (1) 65 years ≤ age ≤ 74 years, (2) 75 years ≤ age ≤ 4 years, and (3) age ≥85 years. The average time between recovery from COVID-19 and surgery was 4.04 weeks (SD, 0.46; range, 1.00–11.00; median, 4.00). Therefore, patients were divided into three groups according to the time between recovery from COVID-19 and surgery: (1) ≤2 weeks, (2) >2 weeks and ≤4 weeks, and (3) >4 weeks. Statistically significant differences were found in terms of age, BMI, ASA classification, time between recovery from COVID-19 and surgery, surgical procedure, and anaesthetic mode between patients with and without postoperative hypoxaemia (*p* < 0.05). Patients with postoperative hypoxaemia had a higher prevalence of expectoration (12.75% vs. 4.93%, *p* = 0.011), preoperative hypoxaemia (32.21% vs. 5.34%, *p* < 0.001), COPD (18.79% vs. 12.34%, *p* = 0.048), and pulmonary inflammation (26.84% vs. 11.11%, *p* = 0.011) than those without postoperative hypoxaemia (Table 1).

No statistically significant differences were found in sex, cough, chest tightness, hypertension, diabetes, coronary heart disease, stroke, smoking status, time from admission to surgery, fracture site, or laboratory parameters (*p* > 0.05) (Tables 1, 2).

**Table 2:** Laboratory parameters of the patients.

	Normal range	Mean ± SD (min, max)	Non-hypoxemia (*N* = 243)	Overall (*N* = 392)	*p*
		Hypoxemia (*N* = 149)			
Leucocytes, ×10^9^ /L	3.5–9.5	5.81 ± 2.44 (2.51–11.17)	5.93 ± 2.12 (2.67–12.11)	5.88 ± 1.99 (2.51–12.11)	0.210
Neutrophils, ×10^9^ /L	2–7.7	4.52 ± 2.75 (1.62–12.93)	4.77 ± 2.34 (1.69–11.99)	4.60 ± 2.21 (1.62–12.93)	0.334
Lymphocytes, ×10^9^ /L	0.80–4.00	0.95 ± 0.55 (0.26–2.27)	0.91 ± 0.33 (0.30–2.16)	0.93 ± 0.39 (0.26–2.27)	0.679
Hemoglobin, g/L	130–175	116.24 ± 12.05 (91–131)	121.01 ± 9.79 (90–147)	119.17 ± 9.98 (90–147)	0.553
Glutamic-pyruvic transaminase, U/L	9–50	26.65 ± 15.98 (8.6–56.4)	27.23 ± 14.76 (9.1–55.9)	27.00 ± 13.99 (8.6–56.4)	0.578
Glutamic-oxalacetic transaminase, U/L	15–40	33.43 ± 24.80 (12.1–94)	32.98 ± 23.16 (12.5–101)	33.17 ± 21.58 (12.1–101)	0.467
Blood urea, mmol/L	3.1–8.0	6.84 ± 4.60 (3.4–18.7)	7.11 ± 4.44 (4.0–15.6)	6.99 ± 3.09 (3.4–18.7)	0.456
Serum creatinine, umol/L	57–97	82.83 ± 23.18 (45.8–136.9)	71.01 ± 24.81 (39.9–141.8)	77.80 ± 15.46 (39.9–141.8)	0.337
Lactic dehydrogenase, U/L	120–250	219.56 ± 34.71 (131–392)	227.34 ± 28.57 (108–338)	222.07 ± 41.21 (108–392)	0.462

Moreover, patients with postoperative hypoxaemia exhibited a lower amount of bleeding (271.80 ± 42.55 vs. 314.80 ± 37.95, *p* = 0.031), fluid infusion volume (1690.70 ± 249.60 vs. 1909.50 ± 245.80, *p* = 0.012) and duration of surgical time (74.65 ± 3.84 vs. 94.52 ± 3.63, *p* = 0.044) than patients without postoperative hypoxaemia. Patients with postoperative hypoxaemia had a longer hospital stay (11.32 ± 1.14 vs. 8.56 ± 2.87, *p* = 0.011) than patients without postoperative hypoxaemia (Table 1).

### 3.4. Multivariate analysis of risk factors for postoperative hypoxaemia

Multivariate analysis showed that a BMI ≥ 28.0 kg/m^2^ [odds ratio (OR) = 1.501, 95% confidence interval (CI) 1.098–3.421, *p* = 0.012], expectoration symptoms (OR = 1.345, 95% CI 1.127–2.908, *p* = 0.001), preoperative hypoxaemia (OR = 2.345, 95% CI 1.442–3.815, *p* < 0.001), ASA classification III (OR = 1.434, 95% CI 1.023–2.010, *p* = 0.037), time between recovery from COVID-19 and surgery ≤2 weeks (OR = 1.695, 95% CI 1.319–2.817, *p* = 0.001), and general anaesthesia (OR = 2.516, 95% CI 1.902–3.897, *p* = 0.018) were independent risk factors for postoperative hypoxaemia (Table 3).

**Table 3:** Multivariate logistic regression analysis for risk factors associated with postoperative hypoxaemia.

Risk factors	Adjusted odds ratio (95%CI)	*p*
BMI (kg/m^2^)		
18.5–23.9	1.0 (reference)	
<18.5	1.718 (0.699–4.223)	0.239
24.0–27.9	1.260 (0.565–5.279)	0.437
≥28.0	1.501 (1.098–3.421)	0.012^*^
Expectoration symptoms	1.345 (1.127–2.908)	0.001^*^
Preoperative hypoxaemia	2.335 (1.442–3.815)	<0.001^*^
ASA classification		
I	1.0 (reference)	
II	1.021 (0.655–1.991)	0.079
III	1.434 (1.023–2.010)	0.037^*^
Time between recovery from COVID-19 and surgery (weeks)		
>4	1.0 (reference)	
2–4	1.776 (0.472–1.977)	0.218
≤2	1.695 (1.319–2.817)	0.001^*^
Anesthetic mode		
Intraspinal anesthesia	1.0 (reference)	
General anesthesia	2.516 (1.902–3.897)	0.018^*^

BMI, body mass index; ASA, American Society of Anesthesiologists; COVID-19, novel coronavirus disease 2019. ^*^p < 0.05.

## 4. Discussion

Severe acute respiratory syndrome coronavirus (SARS-CoV-2) mainly invades respiratory epithelial cells, and patients infected with SARS-CoV-2 can develop an inflammatory response and acute lung injury (16). The symptomatic phase manifests as fever, cough, myalgia, and severe respiratory failure (17). Changes in laboratory parameters include decreased lymphocyte count, increased neutrophil and leukocyte counts, and elevated glutamic-pyruvic transaminase, glutamic-oxaloacetic transaminase, creatinine, and lactic dehydrogenase level (18). Chest CT scans show non-regional ground-glass opacities in the lungs and infiltrative shadows (19). In this study, we found that a certain proportion of elderly patients who had recovered from COVID-19 within 3 months experienced persistent respiratory symptoms, including cough, expectoration, chest tightness, hypoxaemia, abnormal laboratory parameters, and chest CT scan changes, which is consistent with the results of Taquet et al. (20) and Cares-Marambio et al. (21). Taquet et al. (20) reported that, among COVID-19 survivors, 7.90% still exhibited abnormal breathing symptoms and serological indicator changes in the 1 to 180-day period after SARS-CoV-2 infection. Cares-Marambio et al. (21) reported that fatigue, breathlessness, chest pain, and cough were the most common respiratory symptoms of COVID-19 survivors between 3 weeks and 3 months after hospital discharge. However, it is worth noting that respiratory symptoms, such as cough, expectoration, chest tightness, hypoxaemia, and serological indicator changes are not specific to COVID-19 (22). Some patients may develop upper respiratory tract infection and systemic inflammatory response caused by other viruses after recovery from COVID-19 and discharge, which can also cause the above respiratory symptoms and serological changes, leading to an overestimation of the incidence of COVID-19 sequelae.

### 4.1. Incidence of postoperative hypoxaemia

In elderly patients who recovered from COVID-19 and underwent hip fracture surgery in the short term, the incidence of postoperative hypoxaemia was 38.01%, which was higher than the incidence of 30.23% reported in previous studies among non-infected patients and lower than the incidence of 50.00% among patients with COVID-19 (9, 10). In addition, of all postoperative hypoxaemia cases, 92.62% only required mask oxygen inhalation after surgery and 7.38% required mechanical ventilation. Of all the subjects in this study, one patient died due to respiratory failure, one patient died due to pulmonary embolism, and all other patients recovered and were discharged. The mortality rate in this study was much lower than that in previous reports among patients with COVID-19. A study by Catellani et al. (23) reported 13 COVID-positive patients with proximal femoral fractures who underwent surgery in northern Italy, of whom 4 (30.8%) died within 1 week after surgery. Another study by Maniscalco et al. (24), conducted in Italy, reported a mortality rate of 43.8% (14/32) within 21 days after surgery in COVID-positive hip fracture patients. This suggests that elderly patients with hip fractures who have recovered from COVID-19 may have temporarily increased oxygen demands postoperatively, but can safely undergo early surgical intervention after appropriate medical optimisation.

### 4.2. Risk factors for postoperative hypoxaemia

Several studies have shown that obesity is an independent risk factor for postoperative hypoxaemia. Labaste et al. (25) reported that BMI >30 kg/m^2^ was an independent risk factor for hypoxaemia during transfers to PACU in all postoperative patients. Aizawa et al. (26) found that obesity was associated with increased risk of postoperative hypoxaemia, as well as prolonged intubation time and ICU length of stay. In this study, we also found that obesity was an independent risk factor for postoperative hypoxaemia in elderly patients who recovered from COVID-19 and underwent hip fracture surgery. There are two main theories to explain this result: first, in patients with obesity, the decrease in lung compliance is evident, and the work of breathing and respiratory resistance is increased (27); second, obesity is often accompanied by several complications, including obstructive sleep apnoea and hypopnea syndrome, which are related to hypoxia (28).

The incidence of preoperative hypoxaemia is high in patients with hip fracture (13.8–23.8%), and can persist throughout the perioperative period (11, 29). Lung lesions, post-traumatic pulmonary micro thromboembolism, fat embolism syndrome, and damage-associated molecular patterns may contribute to preoperative hypoxaemia (11). In this study, we found that preoperative hypoxaemia was an independent risk factor for postoperative hypoxaemia, which was consistent with the findings of Luna et al. (30). In their study, preoperative hypoxaemia significantly increased the risk of postoperative hypoxaemic events during the first week in patients undergoing joint replacement.

ASA classification system is designed to provide perioperative clinicians with a simple classification of patient physiological status to help predict surgical risk (31). It is used to evaluate the tolerance of elderly patients to anaesthesia before surgery and to accurately predict postoperative cardiopulmonary events (32). In this study, we found that ASA classification III was an independent risk factor for postoperative hypoxaemia. Luna et al. (30) reported that ASA classification had a significant correlation not only with the occurrence of postoperative hypoxemia but also with the occurrence of hypoxemia after discharge.

We also found that general anaesthesia was an independent risk factor for postoperative hypoxaemia, which may be related to postoperative pain, opioid administration, and postoperative muscular strength. A study has shown that for hip surgery, compared with general anaesthesia, patients who received intrathecal anaesthesia had lower pain scores at all evaluated time points and required less postoperative oral morphine (33). Postoperative pain was associated with postoperative hypoxaemia. Compared to patients with severe pain, patients with mild and no pain were 82% and 88% less likely to experience postoperative hypoxaemia, respectively (34). Peripheral tissue oxygen saturation was reduced in response to pain, which may account for the association between severe pain and hypoxemia (35). In addition, general anaesthesia requires muscle relaxants and more opioids than intrathecal anaesthesia. Previous studies have shown that opioids are highly correlated with postoperative hypoxaemia, which is related to the respiratory depression effect of opioids, and that residual neuromuscular blockade is an independent risk factor for critical respiratory events in the PACU (36).

In addition to the above independent risk factors, we found that expectoration symptoms and a time between recovery from COVID-19 and surgery ≤2 weeks were risk factors for postoperative hypoxaemia in elderly patients who recovered from COVID-19 and underwent hip fracture surgery in the short term. Expectoration symptoms can cause hypoxia in patients by blocking the respiratory tract with viscous mucus and sputum (37). In particular, sputum increase during mechanical ventilation under general anaesthesia can further cause airway obstruction (38). Therefore, sputum aspiration should be performed in patients with more sputum during the operation. Moreover, some studies have suggested that the use of a humidifier may be effective if a patient exhibits viscous sputum (39). Lateral position ventilation combined with vibration sputum drainage is more effective in promoting expectoration and improving respiratory function (40). Wirén et al. (41) reported that patients with respiratory disease symptoms had an increased risk of hypoxaemia following surgery. In our study, we found that a high proportion of patients experienced cough, expectoration, hypoxaemia, and pulmonary inflammation within 2 weeks after recovery from COVID-19. This resulted in a higher incidence of postoperative hypoxaemia in elderly patients who have recovered from COVID-19 and underwent hip fracture surgery within 2 weeks.

Univariate analysis showed significant differences in age, COPD, pulmonary inflammation, and surgical procedures between patients with and without postoperative hypoxaemia. However, the logistic multivariate analysis showed that they were not independent risk factors for postoperative hypoxaemia. This may be related to the ASA classification and preoperative hypoxaemia. Patients with advanced age and multiple comorbidities, had a higher ASA classification (42). Unlike THR, HA and PFNA are typically used in elderly and infirm patients with a higher ASA classification (43, 44). Patients with COPD and pulmonary inflammation have a higher risk of developing preoperative hypoxaemia (45).

In addition, we found that patients with postoperative hypoxaemia had a lower amount of bleeding, fluid infusion volume, and duration of surgery than those without postoperative hypoxaemia. This was because a higher proportion of patients in the non-hypoxaemia group underwent THA than those in the hypoxaemia group. Compared with hemiarthroplasty, total HA is complicated, takes a long time, has a large amount of blood loss, and requires a large amount of fluid infusion (46).

Based on the analysis of the above risk factors, we suggest that, it is not advisable to reduce the risk of postoperative hypoxaemia by completely improving respiratory symptoms or by postponing surgery. Research shows that surgical treatment should be performed within the first 24 h, beyond which the odds of perioperative complications increase, and there is a significant increase in mortality when surgery is delayed for more than 48 h (47). Second, in the absence of contraindications, spinal anaesthesia should be administered. General anaesthesia is a risk factor for postoperative hypoxaemia, and all patients who experience severe postoperative hypoxemia received general anaesthesia. Third, for patients who must be administered general anaesthesia, pulmonary function exercise, vibration sputum excretion, and atomisation inhalation can be used to improve pulmonary function, and supplemental oxygen therapy was administered immediately after the operation.

### 4.3. Limitations of this study

This study has two limitations. First, postoperative hypoxaemia was defined as a pulse oxygen saturation of <94% on room air. A variety of factors can interfere with the accuracy of pulse oxygen saturation detection, such as finger cuff displacement, cold fingertip skin, abnormal fingertip skin or colour, and monitoring limb blood oxygen disorders, resulting in low pulse oxygen saturation readings (48). Second, no patients underwent regional block anaesthesia in the study sample. Studies have shown that lumbar plexus block anaesthesia has less impact on circulation and breathing than general anaesthesia and spinal anaesthesia in patients with hip fractures (49–51). Lumbar plexus block anaesthesia may provide greater benefits for older patients who have recently recovered from COVID-19 and undergone hip fracture surgery.

## 5. Conclusion

A certain proportion of elderly patients who recovered from COVID-19 within 3 months still exhibited persistent respiratory symptoms, abnormal laboratory parameters, and chest CT scan changes. In these patients, the incidence of hypoxaemia after hip fracture surgery was 38.01%. Obesity, expectoration symptoms, preoperative hypoxaemia, ASA classification III, time between recovery from COVID-19 and surgery ≤2 weeks, and general anaesthesia were potential risk factors for postoperative hypoxaemia. Our findings suggest that elderly patients with hip fractures who recovered from COVID-19 may have temporarily increased oxygen demands postoperatively but can safely undergo early surgical intervention after appropriate medical optimisation.

## Data availability statement

The data analyzed in this study is subject to the following licenses/restrictions: the datasets used and analysed during the current study are available from the corresponding author on reasonable request. Requests to access these datasets should be directed to JH, haojianhong722@163.com.

## Ethics statement

The studies involving human participants were reviewed and approved by the Institutional Review Coard of HongHui Hospital. Written informed consent for participation was not required for this study in accordance with the national legislation and the institutional requirements.

## Author contributions

JH, WC, and ZL conceived and designed the study, and helped to draft and revise the manuscript. PP, WL, and XL performed data collection. WBC helped to analyse the data and conduct the analysis software. All authors contributed to the article and approved the submitted version.

## Funding

This work was funded by the Key Research and Development Project of Shaanxi Province (No. 2023-YBSF-069) and Xi’an Innovation Capability Strengthening Foundation Program (22YXYJ0019).

## Conflict of interest

The authors declare that the research was conducted in the absence of any commercial or financial relationships that could be construed as a potential conflict of interest.

## Publisher’s note

All claims expressed in this article are solely those of the authors and do not necessarily represent those of their affiliated organizations, or those of the publisher, the editors and the reviewers. Any product that may be evaluated in this article, or claim that may be made by its manufacturer, is not guaranteed or endorsed by the publisher.

## Abbreviations

ASA: American Society of Anesthesiologists
BMI: Body Mass Index
CI: Confidence Interval
COPD: Chronic Obstructive Pulmonary Disease
COVID-19: Coronavirus Disease
CT: Computed Tomography
HA: Hemiarthroplasty
ICU: Intensive Care Unit
OR: Odds Ratio
PFNA: Proximal Femoral Nail Antirotation
SARS-CoV-2: Severe Acute Respiratory Syndrome Coronavirus
SD: Standard Deviation
THR: Total Hip Replacement
